# FiveQ: A new easy‐to‐use validated clinical instrument for tinnitus severity

**DOI:** 10.1111/coa.13973

**Published:** 2022-09-02

**Authors:** James Connell, Ella Harrison, Ahmed Bassiouni, Raguwinder Sahota, Stephanie Laden, Andrew Simon Carney, Andrew Foreman, Suren Krishnan, Sinead O'Brien, John‐Charles Hodge

**Affiliations:** ^1^ Department of Otolaryngology Head and Neck Surgery Royal Adelaide Hospital South Australia Australia; ^2^ Faculty of Health and Medical Science University of Adelaide South Australia Australia; ^3^ College of Medicine and Public Health Flinders University South Australia Australia

**Keywords:** audiology, general, neuro‐otology, tinnitus, quality of life

## Abstract

**Objectives:**

Tinnitus is a complex and debilitating phenomenon with potentially significant implications on quality of life. New presentations can be resource and time intensive for clinicians. Validated comprehensive tinnitus questionnaires may lack practical utility in the high‐volume clinical setting. Concise, targeted questionnaires may offer an efficient alternative. This study aimed to assess the validity of the *FiveQ*, a novel five question construct designed to measure tinnitus severity. Convergent validity was assessed through correlating *FiveQ* against two comprehensive validated questionnaires, the Tinnitus Handicap Questionnaire (*THQ*) and Tinnitus Handicap Inventory (*THI*)*.*

**Design:**

Cross‐sectional study with prospective recruitment. The 117 voluntarily recruited participants completed the *FiveQ*, *THI* and *THQ* questionnaires. Results were comparatively analysed.

**Setting:**

Recruitment was via electronic and print media, audiology clinics and public and private otolaryngology outpatient clinics. Surveys were completed electronically.

**Participants:**

Members of the public aged over 18 with subjective tinnitus were invited to participate.

**Main Outcome Measured:**

Analyses for establishing the content validity, construct validity, internal consistency, explorary factor analysis, and responsiveness of *FiveQ* was performed.

**Results:**

*FiveQ* demonstrated a high positive correlation with both the *THI* (r = 0.773, *p* < .001) and *THQ* (r = 0.808, *p* < .001). Internal consistency for *FiveQ* reached an acceptable threshold (Cronbach's alpha 0.86). Exploratory factor analysis demonstrated that one latent factor underlies the five items of the *FiveQ*. *FiveQ* demonstrated better responsiveness than both the *THI* and *THQ* after a 6 week interval repeat measurement.

**Conclusion:**

*FiveQ* demonstrated high‐positive correlations with existing validated tinnitus questionnaires as well as acceptable internal consistency and factor analysis. The concise construct of *FiveQ* allows clinicians to efficiently estimate tinnitus severity, target treatment towards dominant symptoms and establish a reliable estimation of treatment response following interventions.

**Key Points**

- Many existing validated tinnitus quality of life questionnaires are time intensive and lack practical utility in the busy clinical setting.
- For shorter questionnaires to be effectively integrated into the clinical setting, they must have a concise clinical focus and demonstrate acceptable statistical validity.
- *FiveQ* is a novel five question construct that focuses on patients' core tinnitus symptoms: sleep, mood, hearing, concentration and activities of daily living.
- *FiveQ* demonstrated acceptable internal validity, factor analysis and high‐positive correlations with longer validated tinnitus quality of life questionnaires.
- *FiveQ* is a useful tool that clinicians can adopt as a validated and concise assessment of tinnitus severity, allowing targeted treatment towards the dominant symptom, as well as providing a quick and reliable estimate of treatment response.

## 1 INTRODUCTION

### 1.1 Background and rationale

Tinnitus is the subjective perception of sound in the absence of auditory stimuli.^1^ It represents a complex and debilitating phenomenon that affects one third of individuals throughout their lifetime.^2^ Over 20% of tinnitus sufferers report a severe impairment in quality of life (QOL) as a result of their tinnitus.^3^ Common severe impairments include sleep disturbance, depression, reduced work productivity, social withdrawal or isolation, and hearing loss.^4^

New presentations of tinnitus can be resource and time intensive due to diverse differential aetiologies and wide spectrum of symptomatology. A typical primary care encounter would involve a detailed history, clinical examination, audiogram, investigations, treatment initiation, referral, education and counselling. Associated mood and sleep symptoms invariably necessitate clinicians to diverge into more comprehensive evaluations with an associated impact on consultation time. Establishing an efficient and rapid clinical tool to assess tinnitus severity is fundamental in this setting.^5^

With a range of emerging treatments and no current gold standard,^6^ there is a need for an efficient, digitalised QOL instrument that provides an accurate measurement of severity and is sensitive to treatment related changes.^7^ Established comprehensive questionnaires with multilingual validation, such as the Tinnitus Handicap Questionnaire (*THQ*),^8^ Tinnitus Handicap Inventory (*THI*)^9^ and Tinnitus Functional Index (*TFI*),^10^ play an important role in the accurate assessment of tinnitus. Of those three, only *TFI* is validated for both initial assessment and clinical surveillance. However, the length of these instruments may lack practical application in high‐volume clinical landscapes. Furthermore, tinnitus can fluctuate over time necessitating multiple clinical encounters. The appetite for completing serial time intensive questionnaires may be tempered for patient and clinician. There is evidence that shorter questionnaires achieve greater compliance, both in the hospital and primary care settings.^11^ Within otolaryngology, there is already a trend towards reducing number of questions in QOL instruments such as the Glasgow Benefit Inventory (GBI), with evidence shorter questionnaire can be used without compromising efficacy.^12^

### 1.2 Objectives

Establishing shorter, targeted questionnaires that provide a rapid estimation of tinnitus severity and identify key domains of impairment may offer practical benefits for primary and secondary care clinicians. We set out to determine whether a novel 5‐question questionnaire, *FiveQ*, demonstrated acceptable correlation with validated comprehensive questionnaires, *THI* and *THQ*. Our hypothesis was that individuals who score highly on *FiveQ* would also score highly on longer‐format instruments.

## 2 MATERIALS AND METHODS

### 2.1 Ethics statement

Ethics approval was obtained from the Central Adelaide Local Health Network Human Research Ethics Committee(Approval ID 12796). STROBE reporting guidelines were utilised in the preparation of this research.

### 2.2 Participants

Inclusion criteria were patients aged over 18 with symptomatic tinnitus. Pulsatile tinnitus was excluded. Participation was voluntary and signed consent was obtained. Avenues of recruitment included electronic and print media, non‐for‐profit tinnitus networks, audiology clinics and hospital otolaryngology departments. Each participant was provided with long‐term subscription to the sound therapy app used in this study as gratitude for their participation.

### 2.3 Study design

This was a cross‐sectional study with prospective consecutive patient recruitment. Each participant completed all three questionnaires consecutively in the same sitting to assess the convergent validity of *FiveQ* with validated instruments at baseline. Participants were invited to repeat questionnaires after 6 weeks of sound‐based tinnitus intervention to assess the temporal role and responsiveness of *FiveQ* to changes in symptoms.

### 2.4 Setting

All questionnaires were completed electronically via the web‐based platform SurveyMonkey (*SurveyMonkey Inc*, San Mateo) and the sound‐based tinnitus intervention app.

### 2.5 Validated comparator questionnaires

The *THQ* is a multilingual validated tinnitus QOL instrument containing 27 questions separated into three factors: Factor 1–social, emotional and behavioural; factor 2–tinnitus and hearing; and factor 3‐outlook on tinnitus. Participants self‐report a score from 0 to 100 for each question. Following adjustment for negative questions and weighting, a final score out of 100 is calculated. The *THI* is another validated questionnaire containing 25 questions. Each question has three alternative responses; *yes* (4 points), *sometimes* (2 points), and *no* (0 points). Points are aggregated for a score out of 100. Final scores are graded as slight (0–16), mild (18–36), moderate (38–56), severe (58–76) and catastrophic (78–100).

### 2.6 The *FiveQ* development

*FiveQ* is a short, novel questionnaire developed as a rapid screen for clinicians to estimate and monitor tinnitus severity. An emphasis in content validity for the *FiveQ* development was identifying domains that were likely to undergo clinically important change as a result of treatment.^13^ Development was consumer driven. Engagement of potential users of the questionnaire was initiated early in this process, with tinnitus sufferers recruited from the Office of Ageing database, South Australia. Formal consultation was conducted in person with 19 tinnitus sufferers. During this seminar, consumers identified 10 dominant symptoms. Symptoms were subsequently ranked from 1 to 10 (most important to least important) by both the 19 attending consumers and by a further 22 consumers via an online survey. Based on these results, the top five scoring questions were selected without bias to construct the *FiveQ*.

### 2.7 The *FiveQ* final construct

**FIGURE 1:**
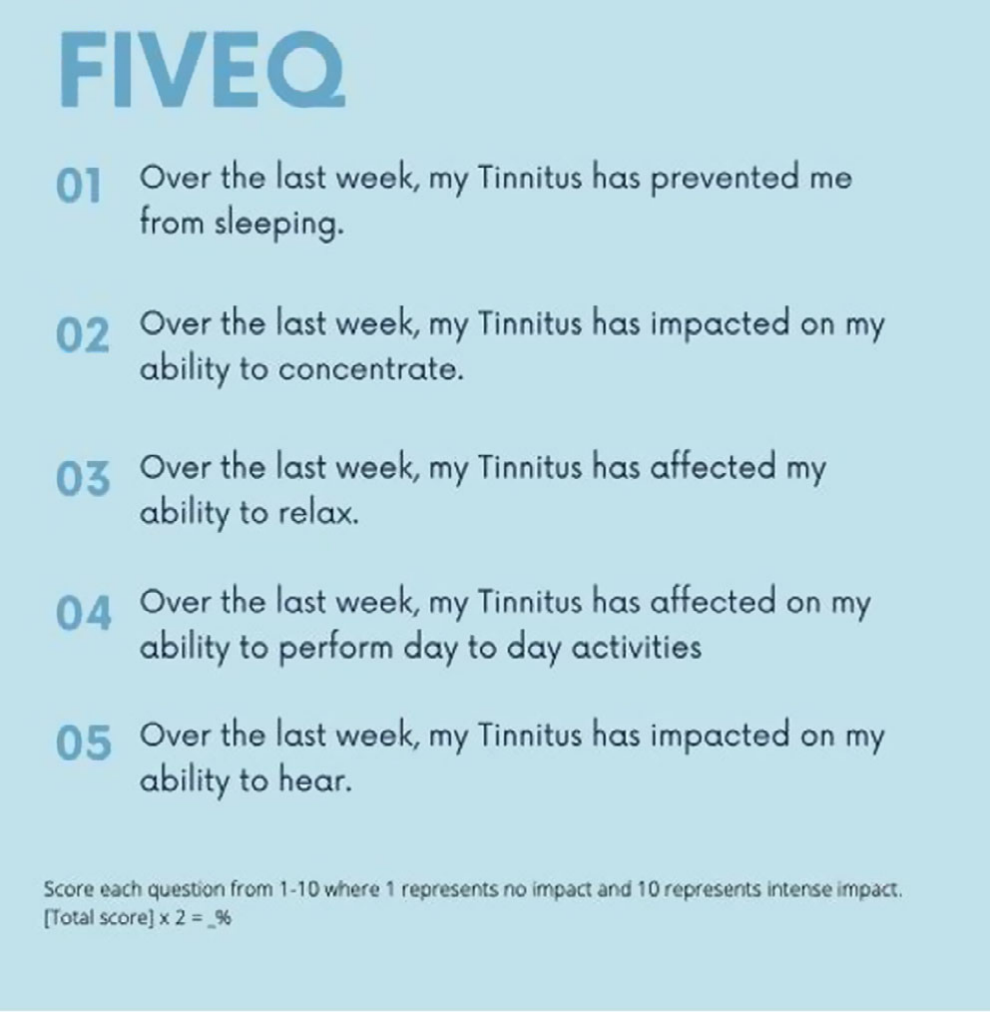
The FiveQ questionnaire contains five questions relating to the key tinnitus symptom domains. Self‐reported symptom severity (over the preceding week) is graded from 1 to 10 where 1 represents no impact and 10 represents a profound or intense impact. A final score is tallied and can be converted to a percentage by (total score) × 2

The five questions examine core tinnitus symptoms of sleep, mood, hearing, concentration and activities of daily living(Figure 1). Patients self‐report a score out of 10 for each question(1 indicates *no impact*, 10 represents *intense impact*). A score out of 50 is multiplied by two to obtain a score out of 100. Grading out of 100 allows for intuitive interpretation by patients and clinician, ease of comparison with similarly graded questionnaires and ease of classification of symptom severity. Similar to comparator questionnaires, bands are in 20% increments, titled slight, mild, moderate, severe and catastrophic. Because tinnitus fluctuates with time, questions are time‐specific, explicitly focusing on the preceding week.

### 2.8 Statistical analysis and outcomes measured

Statistical analyses were performed using *R* (*R Foundation for Statistical Computing*, Vienna, Austria) through the *Jupyter* notebook interface. Detailed statistical methodology is included in supplementary 1. Convergent validity was calculated using Pearson's correlation coefficient(r). Interpretation of the strength of correlations used the terminology by Hinkel.^14^ Cronbach's alpha and McDonald's gamma were calculated to assess internal consistency. The acceptable lower limit for an acceptable Cronbach's alpha was taken at 0.7.^15^ Exploratory factor analysis(EFA) was performed to assess the latent variables or factors underlying the *FiveQ*'s variables. Responsiveness was assessed through measuring the standardised response mean (SRM) across a 6‐week interval, with participants receiving a sound‐based tinnitus intervention in the interim.

## 3 RESULTS

### 3.1 Demographics

Of the 117 patients in the study 32 were male (27%) and 85 female (73%). The median age bracket was 55–64 years, with 72% aged over 55. Bilateral tinnitus was present in 91 (78%), with 26 (22%) reporting unilateral tinnitus only. Tinnitus was single tone in 62 (53%) and multi‐tone in 55 (47%). The average duration of tinnitus symptoms was 12.1 years.

### 3.2 Questionnaire outcomes

Each Questionnaire had a final numeric score of 0–100. The mean scores at baseline (*n* = 117) for all participants were 44.8 (SD = 21.7) for *THI*, 43.4 (SD = 21.7) for *THQ* and 44.41 (SD = 22.1) for *FiveQ*. All results were categorised according to defined severity bands (Table 1). The distribution of participant scores for all three questionnaires were compared using the Kolmogorov–Smirnov test. This test could not reject the null hypothesis that the score distributions of all three instruments were similar (*p* > .05). Six patients (5%) scored the minimum possible *FiveQ* score, while one patient (0.8%) scored the maximum possible score.

**TABLE 1:** Tinnitus severity grading from slight to catastrophic using the *THQ* grading system. *THQ* and *FiveQ* scores out of 100 categorised using the same formula

Grade	*THI*	*THQ*	*FiveQ*
Slight	15	15	20
Mild	35	34	31
Moderate	35	36	34
Severe	16	22	25
Catastrophic	16	10	7

### 3.3 Convergent validity

**FIGURE 2:**
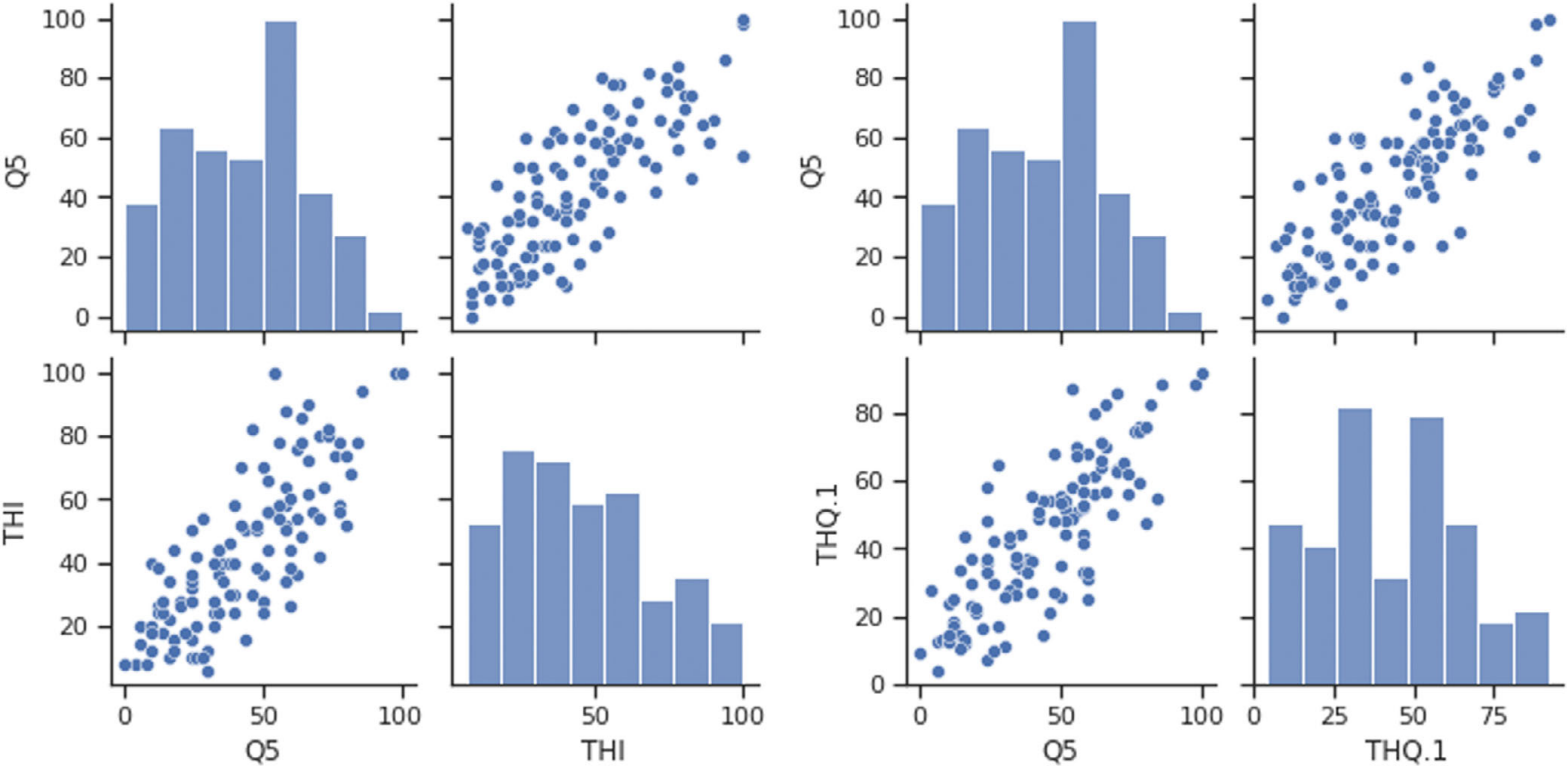
LEFT: Correlation plots between THI and FiveQ (r = 0.808); RIGHT: Correlation plots between THQ and FiveQ (r = 0.773)

Convergent validity of *FiveQ* (*n* = 117) was assessed by determining its correlation with *THI* and *THQ* (Figure 2). *FiveQ* had a high‐positive correlation with *THI* (r = 0.773, *p* < .001) and *THQ* (r = .808, *p* < .001). There was also a high positive correlation between the *THI* and *THQ* (r = 0.855, *p* < .001). Of the 117 participants, 54 repeated all questionnaires after 6‐weeks of sound‐based tinnitus intervention, representing a drop‐off rate of 53.8%. Mean scores after 6‐weeks were *THI* 39.15 (SD = 20.89), *THQ* 41.10 (SD = 20.52) and *FiveQ* 31.54 (SD = 21.44). Pearson's correlations between instruments at 6‐weeks were: r = 0.85, *p* < .001 (*THI* and *THQ*); r = 0.71, *p* < .001 (*FiveQ* and *THI*); and r = 0.70, *p* < .001 (*FiveQ* and *THQ*).

### 3.4 Internal consistency

The correlation matrix (Pearson's correlation coefficient) between the five variables of *FiveQ* is presented in Table 2. Cronbach's alpha and McDonald's omega were calculated to assess the internal consistency of questions within *FiveQ*. For this analysis, the number of observations was 117. The minimum sample size for a Cronbach's alpha analysis for a 5‐item questionnaire(power = 0.80; Type I error = 0.05; null hypothesis value = 0.5) was calculated at 26. This yielded a Cronbach's alpha of 0.86 [CI 0.82–0.9] and McDonald's omega of 0.7. This represents an acceptable value for internal consistency(>0.7 cut‐off).^15^

**TABLE 2:** Pearson's correlation coefficient matrix between each *FiveQ* question

	Activity score	Hearing score	Concentration score	Relax score	Sleeping score
Activity score	1				
Hearing score	0.596	1			
Concentration score	0.680	0.553	1		
Relax score	0.583	0.429	0.811	1	
Sleeping score	0.375	0.296	0.633	0.622	1

### 3.5 Factor analysis

**FIGURE 3:**
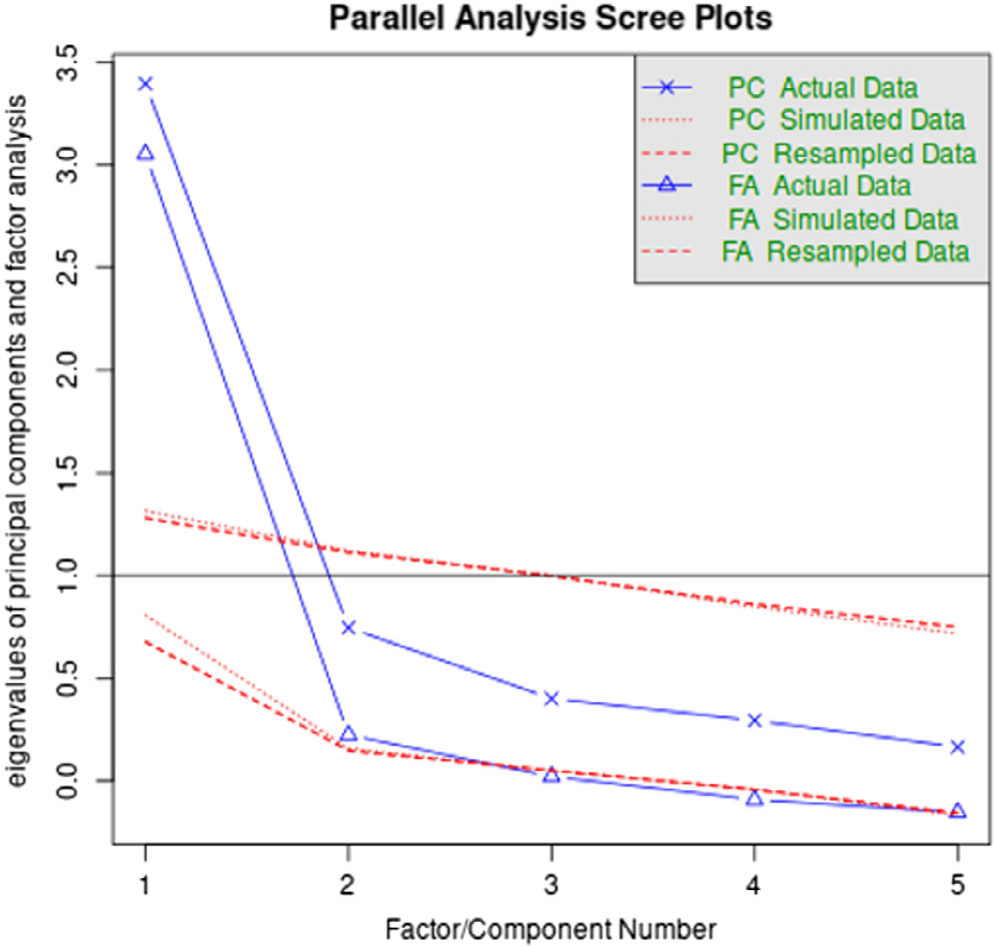
Factor analysis using parallel analysis scree plots demonstrating unidimensional modelling

We analysed whether our data was suitable for factor analysis. The Kaiser‐Meyer‐Olkin (KMO) measure of sampling adequacy was 0.82, suggesting data would be appropriate for factor analysis. Bartlett's test of sphericity suggested there was sufficient significant correlation for factor analysis (Chi‐square = 257.55, *p* < .001). Parallel analysis, Kaiser criterion, and scree plot determined there was one factor underlying the data(Figure 3). Exploratory factor analysis was performed with the number of factors set to 1 and using oblimin rotation method. The total number of observations was 117 with Likelihood Chi Square = 22.08 (*p* < .001), Tucker Lewis Index of factoring reliability = 0.89 and root mean square error of approximation = 0.17. Factor loadings for all variables were greater than 0.5: Activity Score (0.73), Hearing Score (0.59), Concentration Score (0.96), Relax Score (0.84) and Sleeping Score (0.63).

### 3.6 Responsiveness of *FiveQ* and use for symptom follow‐up

Responsiveness of *FiveQ* was assessed through the Standardised Response Mean (SRM). The SRM was calculated as −0.58 (−0.80 adjusted). The interpretation of this value is that the *FiveQ* has a ‘large’ responsiveness index to change(≥0.80 per Cohen).^16^ For comparison, the 6‐week SRM of *THQ* was −0.07 (−0.12 adjusted), and *THI* was −0.28 (−0.45 adjusted). We, therefore, interpret the responsiveness of *THQ* and *THI* questionnaires as trivial and small, respectively. Our results suggest *FiveQ* is potentially more responsive than both *THQ* and *THI* (Table 3).

**TABLE 3:** Responsiveness: 54 out of 117 participants repeated questionnaires after 6 weeks of sound therapy treatment to assess questionnaire responsiveness. FiveQ demonstrated a large responsiveness utilising standardised mean index (SRM). THQ and THI demonstrated trivial and small responsiveness, respectively (Cohen)^16^

	Mean baseline score (*n* = 54)	Mean 6 week score (*n* = 54)	Mean difference (*n* = 54)	Standardised response mean (SRM)
FiveQ	40.67 (SD = 21.52)	31.54 (SD = 21.40)	−9.12 (SD = 15.54)	−0.58 (−0.8 adjusted)
THQ	41.95 (SD = 21.80)	41.10 (SD = 20.52)	−0.86 (SD = 12.34)	−0.07 (−0.12 adjusted)
THI	42.93 (SD = 21.74)	39.15 (SD = 20.88)	−3.78 (SD = 13.25)	−0.28 (−0.45 adjusted)

## 4 DISCUSSION

### 4.1 Main outcomes

We introduced a new 5‐question clinical instrument for assessing tinnitus severity, and we demonstrated that it has adequate internal consistency, is predominantly unidimensional on factor analysis, and has a high‐positive correlation with validated tinnitus questionnaires.

### 4.2 Validated questionnaire outcomes

A high‐positive correlation was observed between *THI* and *THQ* (r = 0.860, *p* < .001). These findings support their ongoing important role in tinnitus management, particularly in the research setting. However, their lengthy construct may render them impractical in the high‐volume setting.

### 4.3 *FiveQ* outcomes

Despite being significantly shorter, the *FiveQ* demonstrated high‐positive correlations with both validated questionnaires (r = 0.773 with *THI* and r = 0.808 with *THQ*) and the distribution of their scores were not dissimilar, as tested by the Kolmogorov–Smirnov test. These findings support *FiveQ* as a sound estimation of tinnitus severity, but in a more succinct and time‐efficient format. Scoring is designed to be simplistic for easy interpretation for patient and clinician. Focusing on core symptoms also allows patients and clinicians to isolate areas of symptomatic change and hone management accordingly. This efficient and focused design can be integrated into the workflow of a busy clinic, to rapidly screen for symptoms and severity, in settings where more comprehensive questionnaires may prove unwieldy.

### 4.4 Responsiveness and longitudinal symptom follow‐up

*FiveQ* is designed to be time‐specific, with users asked to focus on their symptoms within the preceding week. This enables it to be used in symptom follow up. For an instrument to be used as such, it needs to demonstrate an acceptable level of ‘Responsiveness’. Responsiveness has been defined as the ability of the instrument to detect change in the construct being measured. All 117 participants were invited to repeat the questionnaires after 6 weeks of sound‐based tinnitus intervention (described in Methods, and one of the original motivations for developing the *FiveQ*). The 54 participants completed all follow up questionnaires. The outcomes in this subgroup showed the highest correlation coefficient was between the longer format questionnaires(*THI* and *THQ*), however *FiveQ* also maintained high‐positive correlations with *THI* and *THQ*. We assessed the responsiveness of *FiveQ* (through measuring SRM) and found it to display large responsiveness, when compared to *THI* and *THQ*, suggesting that it may be a superior instrument for longitudinal symptom follow‐up.

### 4.5 Long versus short questionnaires

Designing optimal questionnaires is challenging and the verdict on long versus short constructs remains an area of scientific investigation. A meta‐analysis of questionnaires assessing the association of length and response rate found that longer questionnaires had a trend towards a lower overall response rate.^11^ Longer questionnaires provide a more thorough analysis but may compromise on power through low compliance and participation. Where shorter questionnaires correlate strongly with longer questionnaires, the shorter construct may be preferable.^17^ The mean time for participants to complete the *THI* and *THQ* consecutively in our cohort was 7 min. Other commonly used validated tinnitus questionnaires are lengthy in their construct. The Tinnitus Questionnaire and Tinnitus Effects Questionnaire has 52 questions,^18^ the Tinnitus Reaction Questionnaire has 26 questions,^19^ and the *TFI* has 25 questions.^10^ In comparison, *FiveQ* takes less than 1 min to complete. Determining which questionnaire is most appropriate should be tailored to the specific setting. In a high throughput clinical setting, a concise and targeted questionnaire offers advantages, both in initial determination of tinnitus severity as well as follow‐up of treatment efficacy.

### 4.6 Digitalisation of tinnitus QOL questionnaires

The ability to digitalise tinnitus QOL questionnaires onto any media platform is fundamental in determining their usefulness in the clinical and research setting, specifically in follow up of any tinnitus intervention. In this study, *FiveQ* was accessed by patients on a phone‐based App (Apple and Android). This creates digital data sets with minimal administrative effort for subsequent data analysis and research. Digitalisation also has the advantage of reducing missing data because online platforms do not allow users to proceed without completing all questions chronologically. In longer format questionnaires such as the *TFI* missing data is common with up to 10% data reduction deemed acceptable for statistical analysis.^10^

### 4.7 Limitations in *FiveQ* design and future directions

Although we provided evidence for content validity, construct validity and responsiveness for *FiveQ*, it is important to acknowledge the limitations of this study. This could be reflected through applying the COSMIN Checklist against our manuscript (supplementary 2).^20^ The population studied was a focused group that wanted to participate in a sound‐based tinnitus trial. It is unclear how a different tinnitus‐suffering population (for example, those recruited through clinics or hospitals without planned intervention) would alter results and generalisability. Further work is therefore required to evaluate other variables that may affect results and sensitivity to treatment related changes. Ongoing validation will be necessary, both in varied populations, and against other currently validated QOL construct instruments, such as the *TFI*, a recommended instrument for treatment‐related tinnitus changes. We have excluded patients with pulsatile tinnitus (owing to the original motivation of designing the *FiveQ* to assess response to the sound‐based tinnitus intervention app), but there is no contraindication to pulsatile tinnitus using the *FiveQ*, and their inclusion should be reviewed in future studies. Each participant completed one instrument in the one setting, so measures such as test–retest reliability and the related measurement error were not evaluated in this study. The minimal clinically important difference (MCID) for *FiveQ* was not established in this study, and is a focus for future studies. Temporal validation would also benefit from a longer intervention time.

## 5 CONCLUSION

For use in a busy clinical practice, a questionnaire should not only allow an accurate assessment of tinnitus severity, clinically validated, but it should also be delivered quickly to stop patients from becoming fatigued from extensive questions. *FiveQ* is novel, innovative, and demonstrates strong positive correlation with validated questionnaires. Its key benefit over existing constructs is a short, targeted design for practical integration in the busy outpatient setting. This enables practitioners to rapidly estimate overall tinnitus severity and target treatment towards the dominant symptoms. It also provides an estimation of treatment response following intervention with strong correlation to established questionnaires. The correlations and internal consistency borne out of our data endorses *FiveQ* as a practical adjunct for the busy clinician.

## AUTHOR CONTRIBUTIONS

(I) Conception and design: JC Hodge (II) Administrative support: JC Hodge, S O'Brien (III) Collection and assembly: J Connell, E Harrison, S Laden (IV) Analysis and interpretation: A Bassiouni, J Connell, JC Hodge (V) Manuscript writing and editors: All authors (V) Final approval of manuscript: All authors.

## CONFLICTS OF INTEREST

James Connell, Ella Harrison, Ahmed Bassiouni, Raguwinder Sahota, Stephanie Laden, Andrew Simon Carney, Suren Krishnan, Andrew Foreman: No relevant conflict of interest to declare.

John‐Charles Hodge and Sinead O'Brien: Founders of Valetudo Pty Ltd. Valetudo Pty Ltd developed and have a financial interest in the web‐based product ‘TinnitusTreatment’. This product developed and utilises the ‘FiveQ’ questionnaire.

## Supporting information


**Appendix S1** Supporting information

**Appendix S2** Supporting information

## Data Availability

The data that support the findings of this study are available on request from the corresponding author. The data are not publicly available due to privacy or ethical restrictions.
