# Supplementary material for: FiveQ: A new easy‐to‐use validated clinical instrument for tinnitus severity
Source: Clin Otolaryngol. 2022 Sep 2;47(6):672–9. doi: 10.1111/coa.13973 (PMC9826215; doi:10.1111/coa.13973)
Supplement: Supplementary file 1 — Appendix S1 Supporting information [file COA-47-672-s001.docx]

**SUPPLEMENTARY 1 – Statistical Methodology Expanded**

All statistical analyses were performed using *R*(*R Foundation for Statistical Computing*, Vienna, Austria) and the “scipy” Scientific Python package through the *Jupyter* notebook interface. Internal correlations between the scores for the five components of the *FiveQ* were performed using Pearson’s correlation coefficient(r). To assess convergent validity, Pearson’s correlation coefficient was utilized to correlate *FiveQ* scores with *THI* and *THQ* scores. Strength of correlation descriptors used the terminology by Hinkel(1). Cronbach’s alpha and McDonald’s gamma were calculated using the *psych* package, to assess the questionnaire’s internal consistency. Sample size adequacy was performed for Cronbach’s alpha using the Bonnet formula, calculated using the R function ‘sizePOWrel’ in code provided by Bonett(2). The acceptable lower limit for Cronbach’s alpha was taken at 0.7(3).

Exploratory factor analysis (EFA) was performed to assess the latent variables or factors (dimensionality) underlying the *FiveQ*’s variables, using the ‘psych’ R package. Adequacy of factor analysis and determining the number of factors was performed using the ‘check-factor-structure’ and ‘n-factors’ functions respectively, from the “parameters” R package. The Root Mean Square Error of Approximation (RMSEA) and the Tucker Lewis Index (TLI) are two of several EFA fit indices that have been frequently utilized in the literature to assess the goodness of fit of the observed data to the hypothesized model. Recommended cut-off values for these indices as previously suggested were >0.90 (Byrne 1994 (4)) or >0.95 (Hu and Bentler 2009 (5)) for the TLI, and < 0.06 for the RMSEA (Hu and Bentler (5)). These two indices are also used as part of the aforementioned n-factors function to suggest the appropriate number of latent factors underlying the data. For recommended cut-offs of the loadings of questionnaire items onto the underlying latent factor(s) in EFA, Tabachnick and Fidell (2001 (6)) cite 0.32 as the minimum acceptable loading of an item (variable) onto a factor (which equates to approximately 10% overlapping variance with the other variables in that factor).
Tabachnick and Fidell (2001) cite Comrey and Lee (1992 (7)) that loadings in excess of 0.71 are considered excellent, 0.63 very good, 0.55 good, 0.45 fair, and 0.32 poor.
Costello & Osborne (2005 (8)) also suggest that 5 or more strongly loading items (0.50 or higher) are desirable and indicate a solid factor.

Responsiveness of the *FiveQ* was assessed through measuring the standardized response mean (SRM), a widely used statistic to evaluate responsiveness. Responsiveness was evaluated across a 6-week interval, with participants receiving a sound-based tinnitus intervention in the interim. SRM was calculated as the average of differences of scores (6-week score – baseline score), divided by the standard deviation of the differences. For interpretation of the SRM value, we followed the original interpretation of effect sizes by Cohen(9)(trivial<0.20; small 0.20-0.50; moderate 0.50-0.80; large ≥0.80), after applying the adjustment recommended by Middel(10) that accounts for within-subject correlation, to avoid under- or over-estimating the magnitude of change over time.

**Interpreting the Size of a Correlation Coefficient (Hinkle et al)**

| **Size of Correlation** | **Interpretation** |
| --- | --- |
| .90 to 1.00 (−.90 to −1.00) | Very high positive (negative) correlation |
| .70 to .90 (−.70 to −.90) | High positive (negative) correlation |
| .50 to .70 (−.50 to −.70) | Moderate positive (negative) correlation |
| .30 to .50 (−.30 to −.50) | Low positive (negative) correlation |
| .00 to .30 (.00 to −.30) | negligible correlation |

1. Hinkle DE, Wiersma W, Jurs SG. Applied Statistics for the Behavioral Sciences. 5th ed. Boston: Houghton Mifflin; 2003.

2. Bonett DG, Wright TA. Cronbach’s alpha reliability: Interval estimation, hypothesis testing, and sample size planning. Journal of Organizational Behavior. 2015;36(1):3–15.

3. Cortina JM. What is coefficient alpha? An examination of theory and applications. Journal of Applied Psychology. 1993;78(1):98–104.

4. Byrne BM. Testing for the Factorial Validity, Replication, and Invariance of a Measuring Instrument: A Paradigmatic Application Based on the Maslach Burnout Inventory. Multivariate Behav Res. 1994 Jul 1;29(3):289–311.

5. Hu L tze, Bentler PM. Cutoff criteria for fit indexes in covariance structure analysis: Conventional criteria versus new alternatives. Structural Equation Modeling: A Multidisciplinary Journal [Internet]. 1999 Feb 1 [cited 2022 May 19]; Available from: https://www.scienceopen.com/document?vid=f0bb4206-a4db-42f8-a9c7-976040db0769

6. Tabachnick BG, Fidell LS. Using Multivariate Statistics, Fourth Edition. Needham Heights, MA: Allyn & Bacon; 2001.

7. Comrey AL, Lee HB. A First Course in Factor Analysis [Internet]. Psychology Press; 1992 [cited 2022 May 19]. Available from: https://www.taylorfrancis.com/books/9781317844075

8. Costello AB, Osborne J. Best practices in exploratory factor analysis: four recommendations for getting the most from your analysis. 2005 [cited 2022 May 19]; Available from: https://scholarworks.umass.edu/pare/vol10/iss1/7/

9. Cohen J. Statistical Power Analysis for the Behavioral Sciences. 2nd ed. New York: Routledge; 1988. 567 p.

10. Middel B, van Sonderen E. Statistical significant change versus relevant or important change in (quasi) experimental design: some conceptual and methodological problems in estimating magnitude of intervention-related change in health services research. Int J Integr Care. 2002;2:e15.
