# Supplementary material for: FiveQ: A new easy‐to‐use validated clinical instrument for tinnitus severity
Source: Clin Otolaryngol. 2022 Sep 2;47(6):672–9. doi: 10.1111/coa.13973 (PMC9826215; doi:10.1111/coa.13973)
Supplement: Supplementary file 2 — Appendix S2 Supporting information [file COA-47-672-s002.docx]

**SUPPLEMENTARY 2**

**COSMIN Checklist as it applies to manuscript, *FiveQ: a new easy-to-use validated clinical instrument for tinnitus severity.***

| **COSMIN Box** | **FiveQ** |
| --- | --- |
| [A] Internal consistency | - Cronbach's alpha was calculated (Classical Test Theory). - Sample size adequate for Cronbach’s alpha and justification provided. - Unidimensionality of the scale was checked using exploratory factor analysis |
| [B] Reliability | Not Applicable (only a single measurement per participant administered at a time or setting) |
| [C] Measurement error | Not Applicable (only a single measurement per participant administered at a time or setting) |
| [D] Content validity | - Relevancy: patient population (tinnitus sufferers) shared in constructing the items for the FiveQ - Comprehensiveness: only a small number of patients scored the highest or lowest possible score |
| [E] Structural validity | - Exploratory factor analysis was performed |
| [F] Hypotheses testing | - We have formulated hypotheses (a priori) - We provided adequate description provided of the comparator instrument(s) |
| [G] Cross‐cultural validity | Not Applicable (single Australian population) |
| [H] Criterion validity | Not Applicable (no known “golden standard” to measure tinnitus related QoL) |
| [I] Responsiveness | - Two measurements for each participant (one at baseline, another at 6 weeks interval) with intervention in meantime (neuromodulation app use) - Standardized response mean (SRM) reported - Effect of intervention not a topic of this manuscript - Minimally important clinical difference (MCID) not addressed in this manuscript |
| [J] Interpretability | - The distribution of the total scores in the study sample was described - the percentage of participants who had the lowest & highest possible total score was described - The minimal important change (MIC) was not determined. |
| **Generalizability Box** | **Was the sample adequately described in terms of .. ?** |
| 1 median or mean age (with standard deviation or range)? | Yes. |
| 2 distribution of sex? | Yes. |
| 3 important disease characteristics (e.g. severity, status, duration) and  description of treatment? | Yes. |
| 4 setting(s) in which the study was conducted? E.g. general population,  primary care or hospital/rehabilitation care | Yes. General population. |
| 5 countries in which the study was conducted? | Yes. Australia. |
| 6 language in which the HR-PRO instrument was evaluated? | Yes. English. |
| 7 Was the method used to select patients adequately described? e.g. convenience,  consecutive, or random | Yes. Consecutive recruitment. |
| 8 Was the percentage of missing responses (response rate) acceptable? | Yes. |
